# The risk of occupational anaphylaxis in beekeepers: an educational public health intervention

**DOI:** 10.3389/fpubh.2025.1693834

**Published:** 2025-11-25

**Authors:** Tea Močnik, Mihaela Zidarn, Nina Frelih, Sabina Ličen

**Affiliations:** 1 Allergy Unit, University Clinic of Respiratory and Allergic Diseases Golnik, Golnik, Slovenia; 2 Faculty of Medicine, University of Ljubljana, Ljubljana, Slovenia; 3 Faculty of Health Sciences, University of Primorska, Izola, Slovenia

**Keywords:** anaphylaxis, beekeepers, prevention, awareness, education, knowledge, healthcare

## Abstract

**Background:**

Beekeepers are at increased risk for anaphylaxis due to frequent honeybee stings. This study developed an educational program for anaphylaxis prevention and an assessment tool to evaluate the knowledge and attitudes of Slovenian beekeepers.

**Methods:**

The educational program was developed using the Delphi method with 13 healthcare professionals (9 allergists and 4 registered nurses specialized in allergology). The Beekeepers Anaphylaxis Awareness and Learning Questionnaire (BAALQ) consisted of two distinct components, developed by 12 experts. Content validity for the Recognition and Anaphylaxis Action Scale (RAAS) was confirmed by 59 healthcare professionals. Criterion and construct validity were assessed with 143 beekeepers without a history of anaphylaxis, using a nominal scale for measuring knowledge and a 5-point Likert scale for evaluating Socio-Cultural Learning Attitudes Scale (SCLAS). Knowledge differences among beekeepers before and after the educational intervention were evaluated using the Wilcoxon test. Cronbach’s Alpha coefficient was used to assess internal consistency of the instrument.

**Results:**

The RAAS included 26 statements. Kendall’s W was 0.799 (95% CI: 0.718–0.866, *x*^2^ = 359, *p* < 0.001), indicating strong expert agreement, while Cronbach’s alpha was 0.798 (95% CI: 0.756–0.840). To further assess internal consistency, the Split-half method showed a Pearson correlation of 0.733, with a Spearman-Brown correction value of 0.846, confirming the instrument’s good reliability. The Wilcoxon test showed a significant increase in knowledge (*Z* = −10.078; *p* < 0.001), with Mdn scores rising from 18 to 25. The SCLAS included 15 statements. Kendall’s W was 0.714 (95% CI: 0.600–0.814, *x*^2^ = 289, *p* < 0.001). Confirmatory Factor Analysis confirmed three factors: “Self-confidence,” “Importance of knowledge and education,” and “Influence of gender and age.” The model fit well (RMSEA = 0.0618, CFI = 0.925, TLI = 0.911), with Cronbach’s alpha of 0.717 (95% CI: 0.683–0.751).

**Conclusion:**

The developed educational program is effective in improving beekeepers’ knowledge of recognizing and responding to anaphylaxis. The assessment tool shows strong validity and reliability and supports its use in future educational and research contexts.

## 1 Introduction

According to the most recent consensus (1), anaphylaxis is a severe, potentially fatal hypersensitivity reaction that can develop rapidly and affect multiple organ systems. The reaction typically affects the skin or mucous membranes, respiratory, cardiovascular and/or the gastrointestinal system. Life-threatening forms are marked by respiratory distress and/or circulatory instability and can occur even when skin or mucosal symptoms are absent. Timely recognition of anaphylaxis and correct application of epinephrine are life-saving (2).

Hymenoptera venom is the most common cause of fatal anaphylaxis, responsible for about 20% of all such cases (3). In Europe, approximately 200 people die annually from anaphylaxis caused by stings, with Slovenia standing out among countries with the highest mortality rate, with more than 0.55 deaths per million inhabitants annually (4). Beekeepers represent a high-risk group for sensitization and the potential onset of allergic reactions, including anaphylaxis (5–9). A recent Slovenian study reported that 23.7% of beekeepers experienced a first systemic reaction after a honeybee sting (5), while international studies indicate systemic reactions in beekeepers ranging from 14 to 32% (10) and 4 to 26% (11), substantially higher than in the general population (0.5–3.3% in the United States, 0.3–7.5% in Europe) (12, 13). Occupational anaphylaxis data further highlight the increased risk in certain professions. Registered cases show that insects account for the majority of occupational anaphylaxis (82.7%), with beekeepers, gardeners, farmers, and food handlers most often affected. Compared to non-occupational insect anaphylaxis, occupational cases occur more often in younger adults and are more frequently caused by honeybees, underscoring the occupational component of risk (14). Several country-specific studies further illustrate this trend. In Turkey, the prevalence of systemic reactions among beekeepers reached 37.6%, reflecting both the high occupational risk and limited awareness of honeybee venom allergy (9). Among British beekeepers, 21% reported a systemic reaction to bee stings, with more than 2 years of beekeeping before the first reaction identified as an additional risk factor (15). Other factors that have been associated with an increased risk of systemic reactions include receiving fewer than 25 stings per year, experiencing the first stings of the season after a period of inactivity, having a history of atopy, and pre-existing allergic conditions such as rhinitis, conjunctivitis, or asthma when managing hives (16). Allergic beekeepers are advised to cease beekeeping to avoid potentially life-threatening systemic reactions (5). However, as many continue their activity despite the risk, venom immunotherapy should be considered, as it is an effective treatment for hypersensitivity and prevents systemic reactions upon re-exposure to bee stings (5, 17).

The National register of causes of death recorded 27 fatal anaphylaxis cases in Slovenia over a 10-year period, 47% of which were caused by Hymenoptera stings. In nearly all cases, the fatal reaction was the first anaphylactic episode, occurring without any previous episodes. These findings emphasize that insect stings are the primary cause of fatal anaphylaxis in Slovenia, often in individuals with no prior history of hypersensitivity (18). To prevent fatal outcomes of anaphylaxis, it is therefore essential to implement prevention strategies that target individuals without a previous history of anaphylaxis, in accordance with current international guidelines for the management and prevention of occupational anaphylaxis (19). This is further supported by evidence highlighting the importance of education for beekeepers in reducing the risk of systemic reactions (20). The occupational risk of anaphylaxis in beekeepers is at least partially preventable through educational programs that strengthen symptom recognition, promote preventive measures, and improve preparedness for rapid response. Such programs enhance knowledge of insect sting dangers, encourage the use of protective equipment, and ensure correct and timely use of epinephrine auto-injectors in emergency situations. Despite these findings, further research is necessary to define and evaluate effective preventive strategies for occupational anaphylaxis among beekeepers. Addressing this need represents the central aim of the present study.

Existing health education interventions are intended for individuals or groups who have experienced anaphylaxis or are tailored to other subgroups, such as teachers or parents of children with allergies (21–23). Since most beekeepers are stung several times each year, they represent an suitable target group for the development and validation of a preventive strategy. In response, we developed an educational program for beekeepers which we structured into two parts. The first part focused on providing participants with theoretical knowledge about anaphylaxis, its signs, and appropriate management. The second part consisted of practical workshops where participants learned the correct use of adrenaline auto-injector (AAI), including self-administration using a training device. This targeted training is crucial for quick recognition of the symptoms of anaphylaxis and appropriate action in emergency situations, ultimately reducing the risk of fatal outcomes. To evaluate the effectiveness of the educational program, we developed a tailored measurement instrument.

### 1.1 Aim of the study

The objectives of the research are as follows: (1) To develop a health education program for beekeepers, focused on anaphylaxis recognition and the correct use of an adrenaline auto-injector. (2) To develop a measurement instrument to assess the effectiveness of the program, including beekeepers’ knowledge, attitudes, and the social and cultural factors influencing their readiness to learn. (3) To assess the reliability and validity of the developed measurement instrument on a pilot sample of beekeepers. (4) To determine changes in the knowledge and skills of beekeepers who participated in a health education program aimed at preventing anaphylaxis.

## 2 Methods

### 2.1 Study design

**Figure 1:**
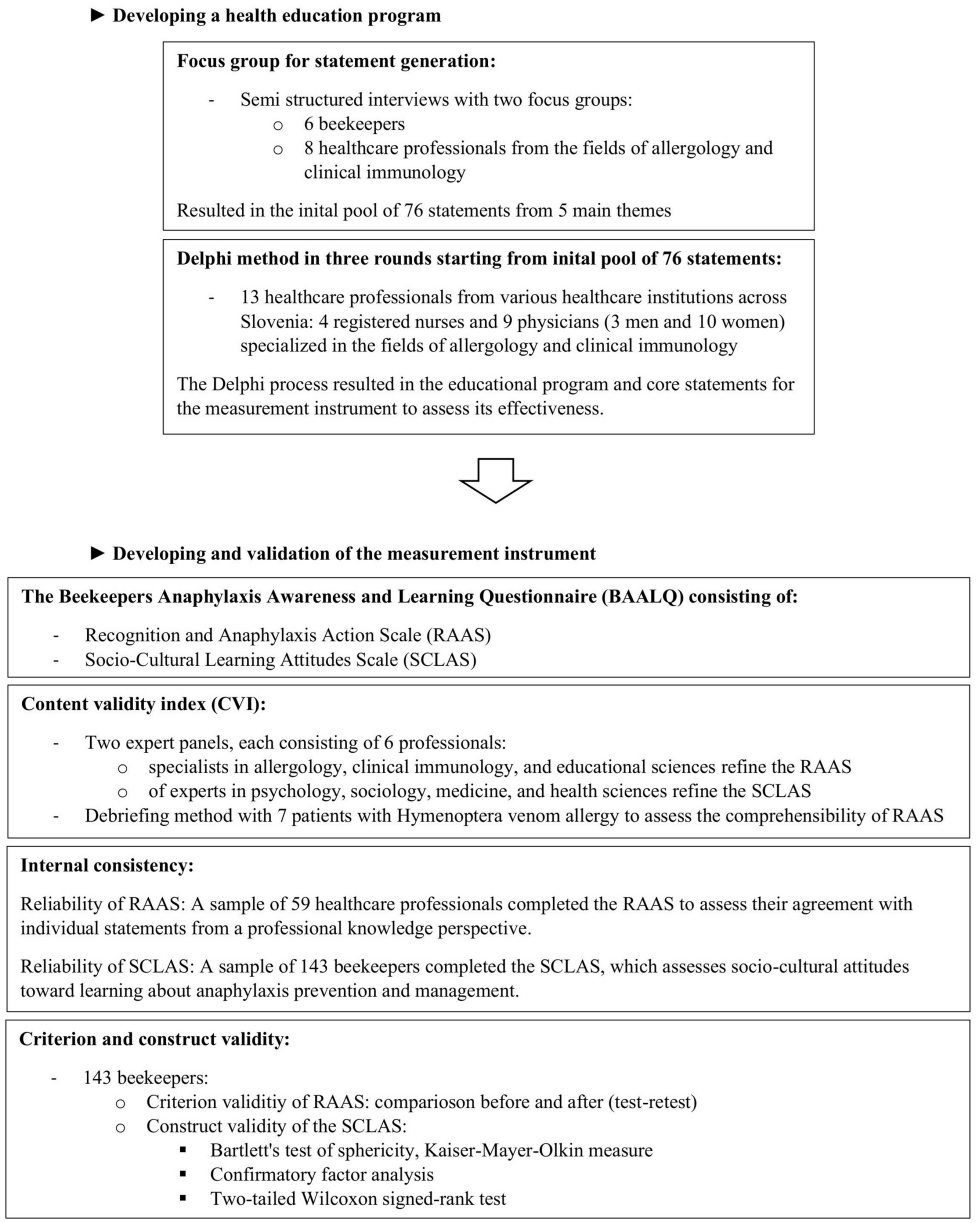
Flowchart of the sequential steps and applied methods in the research process.

In the process of this study, we developed a health education program for increasing the knowledge of beekeepers about the risk of anaphylaxis as a prevention strategy and a measurement tool to assess beekeepers’ knowledge, attitudes, and readiness to learn. The measurement instrument was assessed using multiple validity criteria. We adopted a phased approach that combined qualitative and quantitative methods. For better clarity, the flowchart above (Figure 1) presents the step-by-step research process.

#### 2.1.1 Developing a health education program

**Figure 2:**
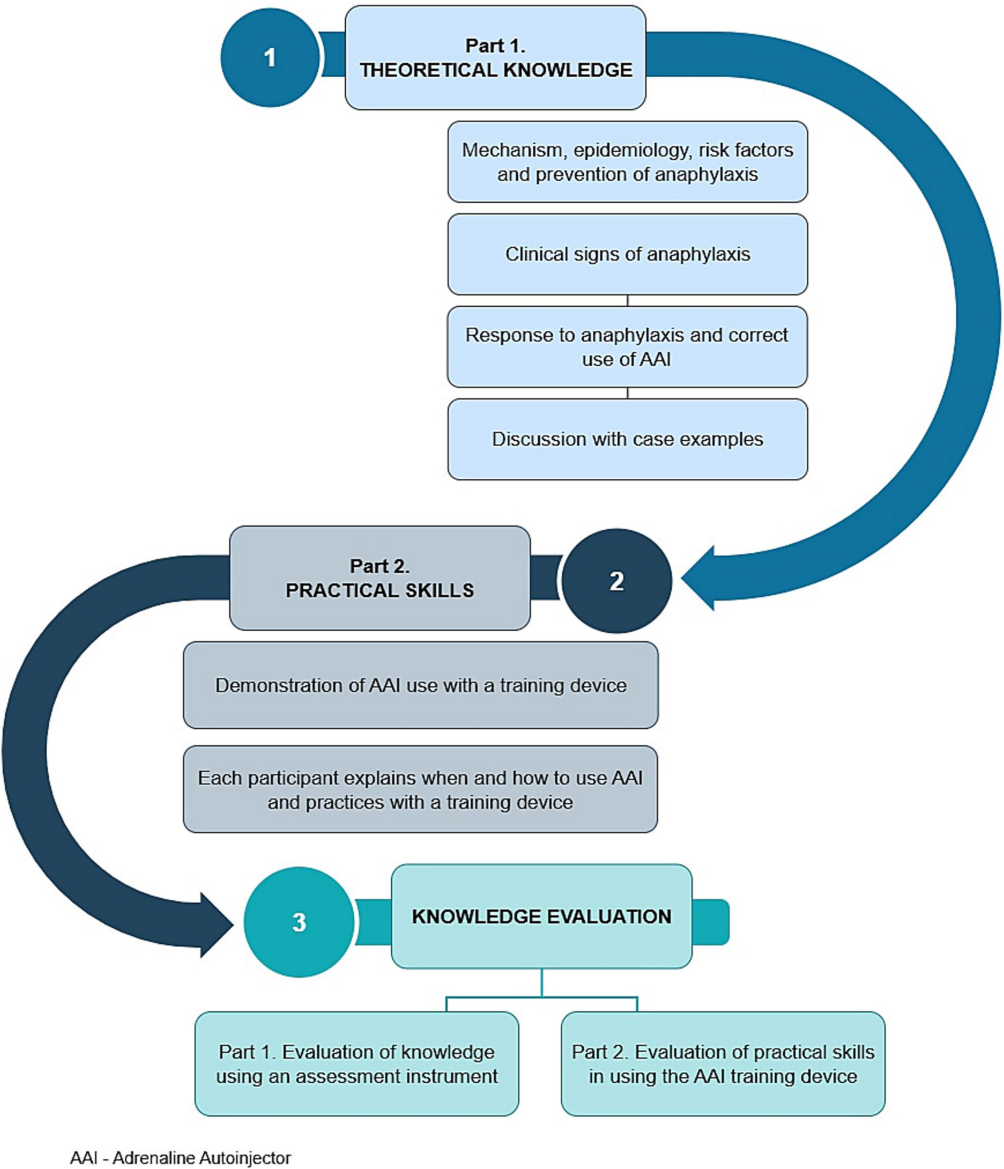
Diagram of the educational process.

The health education program was developed through a qualitative analysis of data obtained from focus groups with beekeepers, and further refined and validated using the Delphi method with a panel of healthcare professionals specialized in allergy and anaphylaxis. The Delphi method was applied according to established methodological guidelines, ensuring a structured process for achieving expert consensus (24, 25). The content and structure of the program are presented in a diagram (Figure 2).

##### 2.1.1.1 Statement generation based on qualitative data

The initial pool of statements was generated based on the results of focus groups. In the study, semistructured interviews were conducted with two focus groups: 6 beekeepers and 8 healthcare professionals from the fields of allergology and clinical immunology (4 men and 10 women). The aim was to examine the cognitive, social, and cultural factors influencing attitudes toward the prevention and management of anaphylaxis, as well as to obtain expert opinions from healthcare professionals. The data were analyzed using thematic analysis, which identified 5 main themes: (1) the management of anaphylaxis; (2) the prevention of anaphylaxis; (3) health education approaches; (4) systemic approaches in prevention; and (5) adrenaline auto-injectors. The results highlighted the need to improve strategies for managing anaphylaxis, the lack of practical educational programs, and the need for greater awareness among beekeepers (26).

##### 2.1.1.2 Delphi method

In the implementation of the Delphi method, for the educational program on anaphylaxis prevention, we included 13 healthcare professionals from various healthcare institutions across Slovenia. The Delphi panel consisted of 4 registered nurses and 9 physicians specialized in allergology and clinical immunology. According to recommendations in the literature, it is advised to include between 10 and 18 experts in a Delphi study to ensure sufficient diversity of opinions while maintaining the manageability of the process (27). The goal of including healthcare professionals from different healthcare centers was to provide a broad range of professional opinions, which enabled greater reliability and validity of the results in the development of the health educational program. In the first round, the experts evaluated statements, critically examined them and provided suggestions for improvements. Based on content analysis of the comments, unclear, irrelevant, or repetitive statements were removed, while the remaining ones were revised and then reviewed again by the experts in the second round to gather further feedback and assess preliminary levels of agreement. In the third round, experts reassessed the revised statements, and those without sufficient consensus were further revised or removed. Participants evaluated statements using the following criteria: (1) clarity (4 = completely clear, 3 = mostly clear, 2 = somewhat clear, 1 = unclear); (2) relevance (4 = completely relevant, 3 = moderately relevant, 2 = low relevance, 1 = irrelevant); (3) importance (5 = very important, 4 = important, 3 = moderately important, 2 = somewhat important, 1 = unimportant).

This structured approach, informed by focus group results and subsequent Delphi rounds, provided a reliable foundation for developing an effective educational program. The Delphi process also served as a basis for formulating the core statements of the Beekeepers Anaphylaxis Awareness and Learning Questionnaire (BAALQ), which was designed to assess the program’s effectiveness (28). Following its development, the questionnaire underwent content validation through expert evaluation, including calculation of Statement-level Content Validity indices. Afterward, reliability testing was conducted to confirm the internal consistency of the instrument, and construct validity was assessed to ensure the questionnaire accurately reflected the underlying theoretical concept.

#### 2.1.2 Development and validation of BAALQ

The developed Beekeepers Anaphylaxis Awareness and Learning Questionnaire (BAALQ) is designed to assess two key components related to managing anaphylaxis among beekeepers: Recognition and Anaphylaxis Action Scale (RAAS), which measures knowledge and response to anaphylactic symptoms, and the Socio-Cultural Learning Attitudes Scale (SCLAS), which assesses socio-cultural attitudes toward learning about anaphylaxis prevention and management. A pilot study was conducted to evaluate the clarity, relevance, and comprehensibility of the preliminary version of the questionnaire.

##### 2.1.2.1 Content validity of BAALQ

Two panels of experts, each consisting of 6 professionals, were involved in evaluating the statements for the development of a BAALQ (with two components RAAS and SCLAS). One panel included specialists in allergology, clinical immunology, and educational sciences, while the other was made up of experts in psychology, sociology, other fields of medicine, and health sciences. Both panels reviewed the statements, which were created based on insights from the Delphi method, focus groups, and a literature review. The Statement-level Content Validity Index (S-CVI) and the Average Method (S-CVI/ave) were calculated to assess the clarity, relevance, and importance of each statement in the questionnaire, with experts determining whether the statements were appropriate for inclusion. In the analysis of the content validity coefficient for each statement based on clarity, relevance, and importance (S-CVI), criteria for assessing the appropriateness of the statements were applied. A threshold value of 0.83 was set for each statement in all three categories (29).

As part of the content validity and clarity of the measurement instrument, we conducted a debriefing method with 7 patients who had hypersensitivity to honeybee venom to assess the clarity of the first part of the RAAS. Cognitive debriefing interviews were conducted in person using the “think-aloud” method, where participants read the statements of the questionnaire aloud and expressed their thoughts and decisions, with each session lasting approximately 30 min per participant (30, 31). Based on the results, we adjusted statements with technical medical terms to improve clarity and usability.

##### 2.1.2.2 Reliability of BAALQ

In the process of assessing reliability during the initial validation of the RAAS, we included a sample of 59 healthcare professionals. We assumed that due to their extensive knowledge, the experts would tend to provide consistent responses to the statements, answering “Yes,” “No,” or “I do not know” which could reduce the variance in responses and negatively affect the internal consistency of the instrument. To optimize internal consistency during the validation phase, we used a 5-point Likert scale (1 = strongly disagree, 5 = strongly agree) for the following reasons: (a) the 5-point scale allowed for a broader range of responses, increasing sensitivity in detecting varying levels of agreement and differences in knowledge among the experts; (b) we assumed that most responses would be very uniform, as the experts were already familiar with the topic. By using a 5-point scale, we reduced the possibility that uniform responses would negatively affect the variance and internal consistency. This allowed for better differentiation between different levels of agreement. To assess the internal consistency of the scales, Cronbach’s alpha coefficient was calculated (32). To further verify internal consistency, we conducted the Split-half method, where we divided the instrument into two halves and then evaluated the correlation between them (33).

For the reliability analysis and calculation of Cronbach’s alpha for the SCLAS, a sample of 143 beekeepers who completed a 5-point Likert scale of agreement (1 = strongly disagree, 5 = strongly agree) was included in the analysis. For pilot testing of the questionnaires, we followed general recommendations from the literature, which suggest a sample size ranging from 30 to 100 participants for this phase (34, 35).

##### 2.1.2.3 Criterion validity of RAAS

In the study, 143 beekeepers completed a RAAS regarding the recognition of symptoms and signs of anaphylaxis and actions taken before and 5 weeks after the education. While expert evaluation employed a 5-point scale to capture agreement levels and optimize internal consistency, the assessment tool for beekeepers used a nominal scale, where the answer “Yes” indicates that the statement is correct, “No” means the statement is incorrect, and “I do not know” expresses uncertainty or lack of knowledge. This simplification was chosen to facilitate straightforward scoring and statistical analysis of knowledge before and after education. Correct answers are awarded points, while incorrect answers or “I do not know” do not earn points, resulting in binary scoring for each item. Binary responses were summed into a total score for each participant. Scores between the two dependent groups, before and 5 weeks after the educational intervention, were compared using the Wilcoxon signed-rank test. Results are presented in the form of medians (Mdn).

##### 2.1.2.4 Construct validity of SCLAS

In the process of analyzing the construct validity of the SCLAS, we included 143 beekeepers who participated in health education program. The SCLAS was designed to go beyond the cognitive aspect of knowledge and provide insight into internal beliefs and social pressure that may influence behavior in critical situations. Respondents rated their agreement with each statement using a 5-point Likert scale (1 = strongly disagree, 5 = strongly agree). On this basis, we established a three-dimensional model of factors, which we empirically tested using Confirmatory Factor Analysis (CFA) to assess how well the proposed measurement structure fits the collected data. Before conducting the analysis, we checked the suitability of the data for this type of analysis using Bartlett’s test of sphericity and the Kaiser-Meyer-Olkin (KMO) measure. With this methodological approach, which ensured that the questionnaire addresses aspects of the target group, thereby ensuring appropriate and targeted data collection, we included a total of 15 statements in the second part of the measurement instrument, covering three factors: self-confidence, the importance of knowledge and education and the influence of gender and age.

##### 2.1.2.5 The final version of the beekeepers anaphylaxis awareness and learning questionnaire

BAALQ, aimed to assess key aspects related to anaphylaxis knowledge, response, and educational readiness among beekeepers, consists of two components: Recognition and Anaphylaxis Action Scale (RAAS) and the Socio-Cultural Learning Attitudes Scale (SCLAS). In line with the approaches of other authors (23, 36), our questionnaire also combines statements that assess actual knowledge and statements that measure attitudes and willingness to participate in education on anaphylaxis prevention. This two-dimensional approach allows for a more comprehensive understanding of the competencies and needs of the target population.

RAAS consists of 26 statements divided into three thematic areas. The first 8 statements focus on recognizing the signs and symptoms of anaphylaxis, with the statements framed as situational descriptions of symptoms and clinical signs that require judgment to determine if they indicate anaphylaxis. The next 6 statements assess knowledge of supportive and additional measures during anaphylaxis. The last 12 statements focus on the use of AAIs, indications for medication administration, proper handling of the device, and further management of the patient. Some statements are deliberately incorrect and serve as control questions to test consistency in responses. The first part of the measurement tool is based on a nominal scale, where the answer “Yes” indicates the statement is correct, “No” means the statement is incorrect, and “I do not know” expresses uncertainty or lack of knowledge on the topic. The analysis of results allows for the assessment of correct recognition of symptoms and signs of anaphylaxis, understanding of key interventions, and proper use of AAIs. Differentiating between correct and incorrect answers, as well as the proportion of “I do not know” responses, enables the statistical evaluation of specific knowledge gaps and identification of areas where additional educational measures are needed. For each correct answer, one point is awarded, while no points are given for incorrect answers or “I do not know” responses. The categorization of nominal variables allows for the quantification of data and statistical analysis based on numerical values.

SCLAS focuses on measuring and assessing beekeepers’ attitudes, confidence, and willingness to learn, as well as social, cultural, and demographic influences. This part consists of 15 statements: 4 focus on self-confidence, 5 on the importance of knowledge and education, and 6 on the influence of gender and age and includes a 5-point Likert scale, allowing for precise measurement of various aspects. The evaluation is based on levels, where 1 means “strongly disagree,” 2 means “disagree,” 3 means “neutral,” 4 means “agree,” and 5 means “strongly agree.” A higher number indicates a stronger agreement with the statement.

### 2.2 Sample

For calculating the S-CVI of the questionnaire, we formed an expert panel comprising 12 professionals (4 men and 8 women; ages ranged from 33 to 63 years) from various fields: allergology, psychology, sociology, and health and educational sciences. Additionally, in this phase, we conducted a debriefing method with 7 patients (4 men, 3 women; mean age: 47.6 years) who had confirmed hypersensitivity to bee venom to assess the comprehensibility of the first part of the questionnaire. Internal consistency was assessed using data from 59 healthcare professionals (32.2% men, 67.8% women; mean age: 45.7 years). Criterion validity and construct validity were evaluated with 143 beekeepers without a history of bee venom allergic reactions (64.3% men, 35.7% women; mean age: 52.8 years). The beekeepers completed the questionnaire to assess its clarity and practical applicability in measuring knowledge, while construct validity was examined through factor analysis within the same sample. Sample sizes were determined based on the questionnaire’s structure and number of items. According to the recommendations of Mundfrom et al. (37), it is generally necessary to obtain at least three participants per statement for conducting factor analysis.

### 2.3 Data collection

Data were collected in close collaboration with the University Clinic for Respiratory and Allergic Diseases Golnik and the Slovenian Beekeepers’ Association, who facilitated access to beekeepers and provided support in conducting the research between November 2024 and March 2025.

### 2.4 Data analysis

The measurement instrument was developed and validated by analyzing the data using MATLAB® and IBM SPSS, version 29.0. Content validity was assessed using the Content Validity Index (CVI), following the recommendations of Polit and Beck (38). For each statement, a CVI was calculated based on expert panel evaluations. Factor and construct validity were assessed using the Kaiser-Meyer-Olkin (KMO) index and Bartlett’s test of sphericity. A KMO value above 0.6 was considered acceptable, and values above 0.8 were considered excellent. Bartlett’s test examined whether the correlation matrix of the data met the assumptions for factor analysis. Criterion validity and differences between dependent groups were assessed using the Wilcoxon test, with a *p*-value < 0.05 considered statistically significant. Internal consistency of the instrument was evaluated using Cronbach’s alpha coefficient, with values above 0.7 considered acceptable (39–42). To provide a conservative and reliable estimate of internal consistency, we additionally calculated the standard error (SE) and 95% confidence intervals (CI) of Cronbach’s alpha using Bonett’s full method (43).

### 2.5 Ethics approval and informed consent

All acquired data are carefully protected in accordance with the Code of Ethics in Healthcare (Official Gazette of the Republic of Slovenia, No. 71/2014), the Personal Data Protection Act (No. 67/07), and the General Data Protection Regulation (EU 2016/679).

Ethical guidelines (World Medical Association, 2013) were followed throughout the research process. The individuals included were not put at risk, as the research was purely theoretical (no samples were collected). All patients were provided with oral and written information on the study and consented to their participation by signing a consent form. The appropriateness of the study has been approved by the Medical Ethics Committee of the Republic of Slovenia (No. 0120-40/2023/6).

## 3 Results

### 3.1 Delphi consensus on the content of the educational program

In the first Delphi round, panel of experts evaluated 76 statements, in the second round 53, and in the third round 44. The results presented in Table 1 refer to the clarity ratings of the 44 statements included in the third round. The low coefficient of variation (CV) values indicate a high level of agreement among experts regarding the clarity of individual statements, meaning the evaluations were consistent. Higher CV values suggest a wider range of evaluations, indicating greater heterogeneity in the experts’ opinions on certain aspects of the statements.

**Table 1:** Assessment of statement consistency regarding educational program content in the third Delphi round.

Statement	Clarity			Relevance			Importance		
	*M*	SD	CV (%)	*M*	SD	CV (%)	*M*	SD	CV (%)
S1 Basic understanding of anaphylaxis and immunological mechanisms	3.85	0.38	10	3.46	0.52	15	4.69	0.48	10
S2 Understanding the risk of anaphylaxis in beekeeping	4.00	0.00	0	4.00	0.00	0	5.00	0.00	0
S3 Awareness of risk factors for anaphylaxis	4.00	0.00	0	3.92	0.28	7	4.85	0.55	11
S4 Knowledge of the stages of systemic hypersensitivity reactions	3.77	0.60	16	3.62	0.65	18	4.31	1.03	24
S5 Ability to recognize symptoms and signs of anaphylaxis	4.00	0.00	0	4.00	0.00	0	4.92	0.28	6
S6 Differentiating between health problems caused by Hymenoptera stings and other unrelated problems	3.77	0.44	12	3.62	0.51	14	4.31	0.75	17
S7Awareness of toxic reactions resulting from multiple stings	3.92	0.28	7	3.85	0.38	10	4.46	0.78	17
S8 Knowledge of factors that may worsen the condition during anaphylaxis	3.77	0.44	12	3.62	0.51	14	4.23	0.73	17
S9 Response to large local reactions	4.00	0.00	0	3.69	0.48	13	4.31	0.95	22
S10 Knowledge of medications to reduce major swelling after stings	3.92	0.28	7	3.54	0.52	15	4.00	1.22	31
S11Knowledge and skills in providing first aid and basic resuscitation procedures	4.00	0.00	0	4.00	0.00	0	4.92	0.28	6
S12 Knowledge of medications for treating mild systemic hypersensitivity reactions	4.00	0.00	0	3.85	0.38	10	4.62	0.65	14
S13 Knowledge of medications for treating anaphylaxis	4.00	0.00	0	4.00	0.00	0	5.00	0.00	0
S14 Basic understanding of the main effects of adrenaline in anaphylaxis treatment	3.85	0.38	10	3.69	0.48	13	4.38	0.77	18
S15 Awareness of possible side effects in anaphylaxis treatment with adrenaline	3.92	0.28	7	3.77	0.44	12	4.31	0.85	20
S16 Knowledge and skills for proper activation of an AAI	4.00	0.00	0	4.00	0.00	0	5.00	0.00	0
S17 Understanding correct storage and maintenance of the AAI	4.00	0.00	0	4.00	0.00	0	5.00	0.00	0
S18 Handling a used AAI	3.92	0.28	7	3.69	0.48	13	4.38	0.65	15
S19. Ability to recognize situations requiring administration of medication via AAI	3.92	0.28	7	4.00	0.00	0	5.00	0.00	0
S20 Knowledge of mistakes and consequences of errors in handling the AAI	4.00	0.00	0	4.00	0.00	0	5.00	0.00	0
S21 Knowledge of additional measures during anaphylaxis	4.00	0.00	0	4.00	0.00	0	5.00	0.00	0
S22 Understanding and implementing preventive measures to avoid anaphylaxis	4.00	0.00	0	3.85	0.38	10	4.69	0.75	16
S23 Ensuring safety for oneself and others involved in beekeeping	4.00	0.00	0	3.77	0.44	12	4.54	0.78	17
S24 Ability to maintain and recognize signs of wear or damage on protective equipment	3.92	0.28	7	3.69	0.48	13	4.46	0.78	17
S25 Responsibility in working with bees	3.92	0.28	7	3.69	0.48	13	4.69	0.48	10
S26 Ability to quickly decide when and how to activate emergency services	3.92	0.28	7	4.00	0.00	0	5.00	0.00	0
S27 Preparedness to act when immediate medical help is not available within minutes	4.00	0.00	0	4.00	0.00	0	5.00	0.00	0
S28 Ability to cooperate with emergency services and rescue teams	3.92	0.28	7	3.92	0.28	7	4.92	0.28	6
S29 Knowledge of procedures for informing the emergency dispatcher	3.92	0.28	7	3.77	0.44	12	4.77	0.44	9
S30 Basic understanding of the purpose of health education	3.85	0.38	10	3.85	0.38	10	4.62	0.51	11
S31 Ability to use different educational tools and techniques for learning	3.77	0.44	12	3.69	0.48	13	4.15	0.80	19
S32 Basic awareness of the latest findings in anaphylaxis prevention	3.77	0.44	12	3.62	0.51	14	4.15	0.90	22
S33 Ability to adopt preventive approaches and motivation to manage and prevent anaphylaxis	3.85	0.38	10	3.85	0.38	10	4.46	0.52	12
S34 Ability to motivate other beekeepers to implement preventive measures for anaphylaxis management	3.92	0.28	7	3.92	0.28	7	4.46	0.52	12
S35 Understanding the need for health education in beekeeping	3.92	0.28	7	3.85	0.38	10	4.46	0.66	15
S36 Ability to accept suggestions from health institutions for progress in anaphylaxis management	3.77	0.44	12	3.46	0.78	22	3.92	1.12	28
S37 Ability to report and document events related to anaphylaxis	3.92	0.28	7	3.92	0.28	7	4.62	0.51	11
S38 Understanding reports and recommendations related to anaphylaxis events	3.54	0.52	15	3.62	0.51	14	4.08	0.86	21
S39 Ability to maintain and update documentation in line with preventive education	3.54	0.52	15	3.62	0.51	14	3.92	0.76	19
S40 Understanding the importance of conducting regular evaluations and updates of preventive measures	4.00	0.00	0	3.92	0.28	7	4.38	0.65	15
S41 Motivation to acquire new knowledge, improve skills, and refresh knowledge and skills	4.00	0.00	0	3.92	0.28	7	4.77	0.44	9
S42 Managing stress during emergency situations	4.00	0.00	0	4.00	0.00	0	4.77	0.44	9
S43 Ability to communicate clearly with healthcare professionals or emergency medical services	4.00	0.00	0	4.00	0.00	0	4.85	0.38	8
S44 Ability to seek and accept professional psychological help if needed	3.92	0.28	7	3.92	0.28	7	4.31	0.63	15

The Kendall’s W test showed statistically significant agreement among the experts (*p* < 0.001), for clarity 0.172, relevance 0.200, and importance 0.297, indicating that the opinions were partially aligned, but there was considerable variation in the assessments among the experts. At this stage, statements were not yet removed solely based on the coefficient of variation or the Kendall’s W coefficient, as these indicators are mainly used to analyze the level of consensus.

### 3.2 Content validity of the BAALQ

The S-CVI/ave. for individual categories of RAAS were as follows: clarity 0.93, relevance 0.90, and importance 0.84. These values indicate strong evaluator agreement, confirming the statements’ appropriateness based on all three criteria. A total of 41 statements of RAAS were evaluated, and those not meeting the S-CVI threshold were modified or excluded, leaving 30 statements after content validity. After removal, Kendall’s W coefficient was 0.799 (95% CI 0.718–0.866, *x*^2^ = 359, *p* < 0.001), showing high expert agreement in statement evaluation.

The S-CVI/ave. for the individual categories of SCLAS were for clarity 0.96, relevance 0.96, and importance 0.97, indicate that the participants expressed a very high level of agreement across all three dimensions for the addressed area. A total of 30 statements were initially evaluated, and those not reaching the threshold value of 0.83 for the S-CVI were revised or excluded, leaving 27 statements after content validity (29). After excluding the statements that did not reach consensus, Kendall’s W was 0.714 (95% CI: 0.600–0.814, *x*^2^ = 289), indicating a high level of agreement among the experts in evaluating the statements.

### 3.3 Internal consistency test–retest reliability of RAAS

For the reliability analysis of the RAAS, a Cronbach’s alpha of 0.798 (95% CI: 0.756–0.840) was achieved in the health professionals’ group, indicating good internal consistency of the scale within this group (32). The results showed a Pearson correlation of 0.733, indicating good reliability of the instrument. Using the Spearman-Brown correction, we obtained a value of 0.846, which further confirms the reliability of the instrument. Based on content validity and reliability, we retained the 26 statements in the first part of the measurement instrument intended for knowledge evaluation. For the SCLAS, the Cronbach’s alpha result of 0.717 (95% CI: 0.683–0.751) indicates acceptable internal reliability of the scale.

In the study, the test–retest was conducted on beekeepers who completed the RAAS before and 5 weeks after the education. Characteristics of the group are presented in Table 2.

**Table 2:** Demographic characteristics of beekeepers.

Category	Subcategory	Value (%/ave)
Gender	Male	64.34%
	Female	35.66%
Age (average)	–	52.77 years
Education	Primary school	1.40%
	Vocational/secondary	37.06%
	University	48.25%
	Postgraduate	9.79%
Beekeeping years (average)	–	14.28 years
Bee stings per season	<5 per season	17.48%
	5–20 per season	23.08%
	>20 per season	48.25%
	Do not know/do not track	9.09%

**Figure 3:**
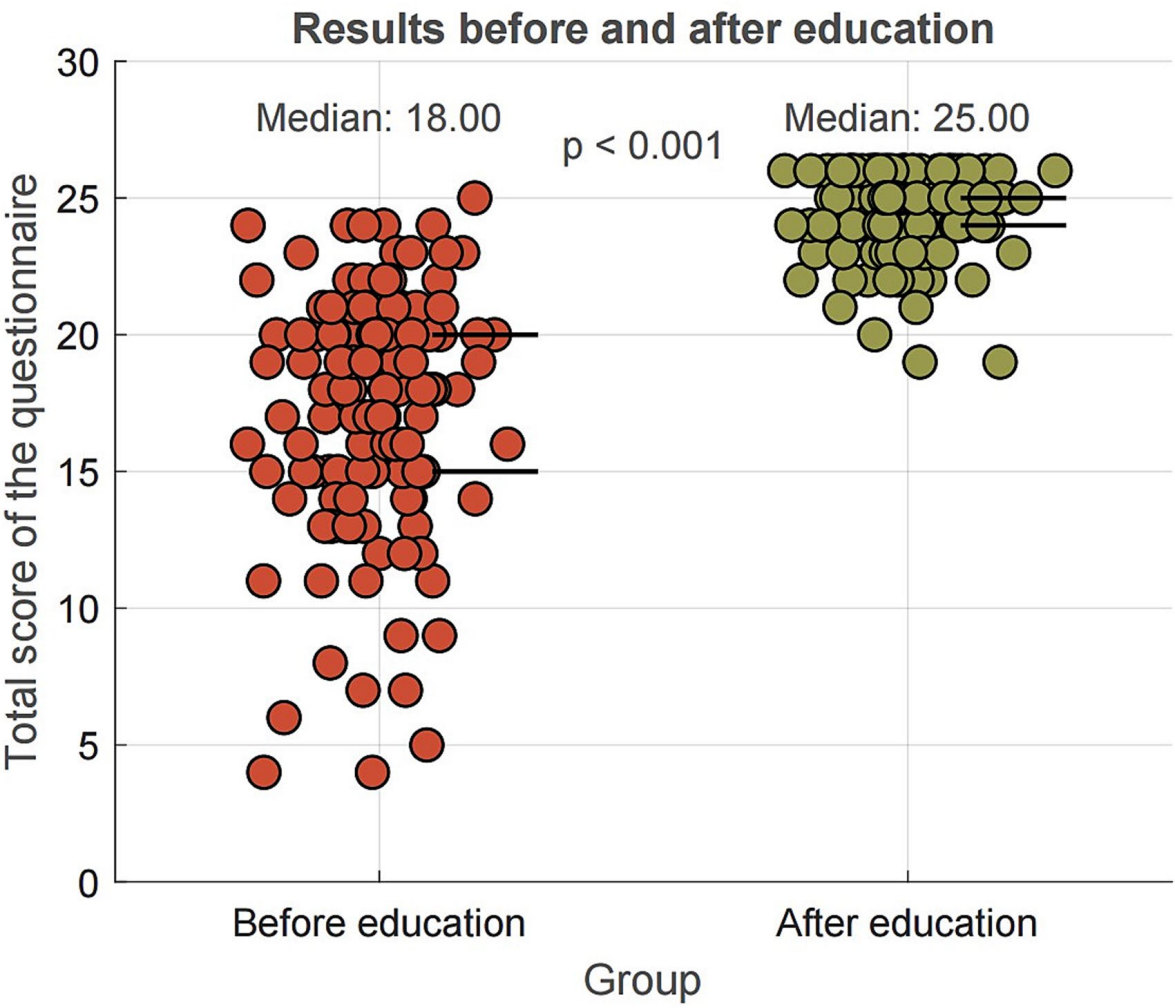
Comparison of pre- and post-education questionnaire scores.

The results showed a statistically significant difference (*Z* = −10.078; *p* < 0.001) in the median scores of the questionnaire between the pre-education group (Mdn = 18, IQR: 15.0–20.0) and the post-education group (Mdn = 25, IQR: 24.0–25.0), indicating significant changes in correct and incorrect answers in the two observed groups (Figure 3). Following the educational intervention, participants’ median total score increased by 7 points (SE = 0.38; 95% bootstrap CI: 6.00–8.00), reflecting a statistically significant improvement. The narrow confidence interval and low standard error indicate that this estimate of the median difference is precise and unlikely to be due to chance.

Similarly, when evaluating practical skills, a significant improvement was observed in the participants’ ability to correctly use the AAI on the training device, with 95.1% of participants successfully demonstrating all steps of the technique within the required time frame of 1 min.

### 3.4 Construct validity of SCLAS

To assess the construct validity of the SCLAS, Bartlett’s test indicated that the data are suitable for factor analysis (*x*^2^ = 699, df = 105, *p* < 0.001), while the Kaiser-Meyer-Olkin test achieved a value of 0.762, suggesting that the data are appropriate for further investigation. From the performed Confirmatory Factor Analysis (CFA), we confirmed three main factors: “Self-confidence,” “Importance of knowledge and education,” and “Influence of gender and age.” All factor loadings were statistically significant (*p* < 0.001), indicating that all included indicators significantly contributed to all three factors. Although several statements exhibited factor loadings below the conventional threshold of 0.30, they were retained because decisions regarding item removal were based not solely on loading values but also on other diagnostic criteria, including theoretical relevance and the overall measurement model fit (Table 3) (44). The RMSEA was 0.0618, which is within the acceptable range (less than 0.08), meaning the model fits the data. The CFI was 0.925 and TLI was 0.911, indicating a very good fit of the model to the data, as both values are above the threshold of 0.90. These results indicate that the model is appropriately fitted to the data and that the confirmed factors are statistically relevant and meaningful in the context of the research area. The bootstrap analysis (10,000 resamples) produced factor loadings and confidence intervals that were generally in line with the CFA estimates, particularly for items with stronger loadings, supporting the stability of the factor structure (Table 4). Some items with lower CFA loadings showed wider confidence intervals, indicating less stable contributions to their respective factors.

**Table 3:** Results of confirmatory factor analysis for the SCLAS.

Factors	Statements SCLAS	Factor loadings	SE	*z*-value	*p*-value
1. Self-confidence regarding the ability to recognize symptoms of anaphylaxis and take appropriate action	1b. I am confident that I can recognize the symptoms and signs of anaphylaxis in myself at this moment	0.465	0.0516	9.01	<0.001
	2b. I am confident that I can recognize the symptoms and signs of anaphylaxis in someone else at this moment	0.370	0.0519	7.14	<0.0001
	3b. At this moment, I am proficient in using an AAI if I need to inject it to myself	0.174	0.0325	5.36	<0.001
	27b. If I were regularly educated about recognizing the symptoms and signs of anaphylaxis, I would feel more confident in taking action	0.187	0.0484	3.86	<0.001
2. Importance of knowledge and education	8b. I believe that every beekeeper should undergo training on recognizing the symptoms and signs of anaphylaxis and how to respond	0.255	0.0291	8.75	<0.001
	9b. Knowledge of recognizing the symptoms and signs of anaphylaxis and how to respond would help me increase safety in my work as a beekeeper	0.240	0.0251	9.56	<0.001
	10b. believe that beekeepers are not fully aware of the dangers of anaphylaxis	0.215	0.0772	2.78	0.005
	11b. I believe that education on recognizing the symptoms and signs of anaphylaxis and how to respond is crucial for the safety of beekeepers	0.148	0.0293	5.03	<0.001
	14b. I am actively seeking information on recognizing the symptoms and signs of anaphylaxis and how to respond	0.260	0.0798	3.26	0.001
3. Influence of gender and age on the perception of anaphylaxis risk and the importance of training	15b. I believe that female beekeepers are more aware of the risk of anaphylaxis than male beekeepers	0.763	0.0901	8.47	<0.001
	17b. Male beekeepers rely more on their own experiences than on formal education about recognizing the symptoms and signs of anaphylaxis and how to respond	0.860	0.0787	10.94	<0.001
	18b. Female beekeepers more frequently seek additional information about recognizing the symptoms and signs of anaphylaxis and how to respond than male beekeepers	1.038	0.0804	12.91	<0.001
	24b. Younger beekeepers are more willing to learn about recognizing the symptoms and signs of anaphylaxis and how to respond than older beekeepers	0.396	0.1017	3.90	<0.001
	25b. In my environment, there is a prevailing opinion that female beekeepers should be more aware of health risks, such as anaphylaxis, compared to male beekeepers	0.383	0.0641	5.98	<0.001
	26b. In the beekeeping community, male beekeepers are more likely to take responsibility for responding in emergency situations like anaphylaxis, compared to female beekeepers	0.363	0.0769	4.72	<0.001

**Table 4:** Bootstrap analysis of standardized factor loadings and 95% CI (10,000 resamples).

Factor	Statement of SCLAS	Average Loading	95% CI lower	95% CI upper
Factor 1	Statement 1b	0.8668	0.6751	0.9975
	Statement 2b	0.6421	0.4921	0.8076
	Statement 3b	0.4646	0.2899	0.6236
	Statement 27b	0.3536	0.1705	0.5428
Factor 2	Statement 8b	0.7433	0.3940	0.9975
	Statement 9b	0.8073	0.4833	0.9975
	Statement 10b	0.2569	0.0858	0.4675
	Statement 11b	0.4666	0.1462	0.7739
	Statement 14b	0.2890	0.0818	0.4448
Factor 3	Statement 15b	0.6584	0.5067	0.8026
	Statement 17b	0.8005	0.6964	0.8933
	Statement 18b	0.9090	0.8352	0.9828
	Statement 24b	0.3365	0.1573	0.4949
	Statement 25b	0.5007	0.3272	0.6726
	Statement 26b	0.4062	0.2064	0.6013

To assess the construct validity of the factors, a two-tailed Wilcoxon signed-rank test was conducted to test whether the factor scores differed significantly from the median (Mdn = 3) under the null hypothesis. The results demonstrated statistically significant deviations for all three factors. Beekeepers considered themselves confident in recognizing anaphylaxis signs and using the AAI after the educational program (Factor 1: Mdn = 5; IQR: 4.5–5.0; 95% bootstrap CI: 5.0–5.0; *Z* = 10.6871; *p* < 0.001). They also regarded anaphylaxis education as important (Factor 2: Mdn = 5; IQR: 5.0–5.0; 95% bootstrap CI: 5.0–5.0; *Z* = 11.4559; *p* < 0.001). In contrast, participants reported that gender and age did not influence their perception of risk or their engagement with the training (Factor 3: Mdn = 2; IQR: 1.0–3.0; 95% bootstrap CI: 2.0–2.0; *Z* = −8.6811; *p* < 0.001). The narrow 95% CI indicate that the median estimates were consistent within the sample, confirming that beekeepers generally felt confident in handling anaphylaxis, valued the education, and considered that gender and age did not affect their engagement.

**Figure 4:**
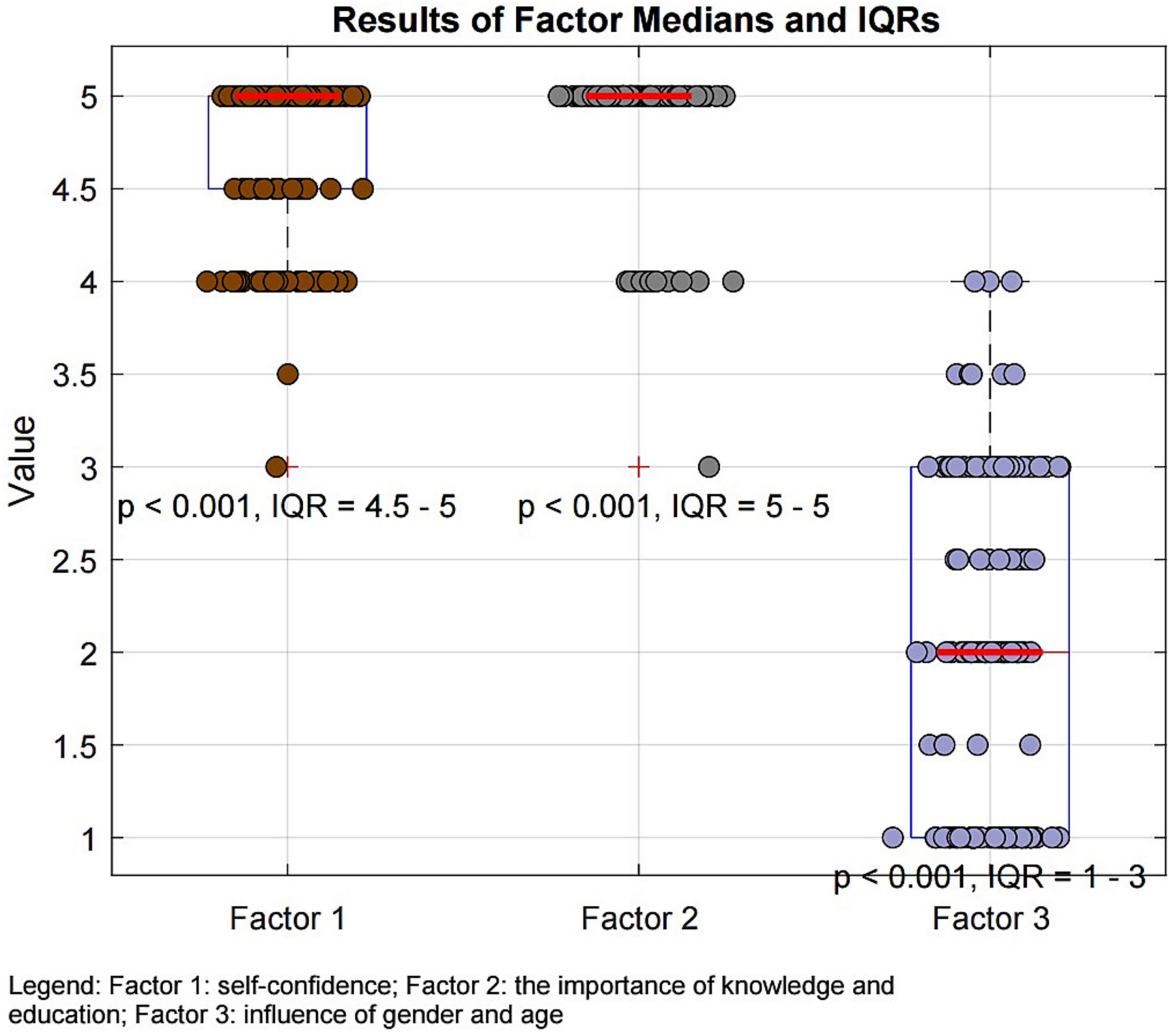
Levels of beekeepers’ self-confidence, knowledge, and perceptions of gender and age influences on learning and action.

The median (Mdn) and interquartile range (IQR) for self-confidence (Factor 1), the importance of knowledge and education (Factor 2), and the influence of gender and age (Factor 3) are presented in Figure 4.

Since knowledge in this case is a non-latent construct that can be directly measured, factor analysis was not performed for the RAAS. A latent construct refers to variables that cannot be directly observed (such as attitudes or beliefs), whereas knowledge is an observable and measurable variable (45).

## 4 Discussion

The Beekeepers Anaphylaxis Awareness and Learning Questionnaire (BAALQ) assesses both beekeepers’ knowledge of anaphylaxis and their social and cultural readiness to manage related risks, providing valuable insights into the effectiveness of the educational program and guiding further interventions to improve anaphylaxis prevention. The validity and reliability analysis demonstrated that BAALQ is well-suited for evaluating these dimensions. The instrument shows strong internal reliability and validity, while confirmatory factor analysis confirmed the suitability of the three-dimensional model used to capture the intended constructs. Although this questionnaire was developed and validated specifically for Slovenian beekeepers, focusing on anaphylaxis risk from honeybee stings, its structure and content could be relatively easily adapted for other occupational groups at increased risk of insect stings. Adaptation for other types of allergies, such as food or environmental allergens, would require more extensive modifications. If this questionnaire is implemented in other countries, cultural and linguistic adaptations would be necessary to ensure that the items are relevant and understandable in different contexts. Future research could focus on cross-cultural validation to assess the applicability and reliability of the instrument in diverse settings.

The results of the study confirm that a structured educational program on anaphylaxis significantly contributes to improving beekeepers’ knowledge. The statistically significant increase in the median knowledge scores after the education highlights the effectiveness of the program in strengthening key skills for recognizing and managing anaphylactic reactions. While this program is primarily educational, it can also contribute to the primary prevention of occupational anaphylaxis, although its full preventive effect is not yet confirmed.

Administering an AAI during anaphylaxis may be challenging without prior instruction, thus necessitating appropriate training to ensure correct use (46). Timely administration can prevent severe complications and provides crucial time until the arrival of emergency medical assistance (47). Sasaki et al. (48) showed that practical workshops for school personnel, including hands-on training with an AAI, significantly improved participants’ self-efficacy in managing anaphylaxis, highlighting the importance of practical skills for both confidence and competence. Similarly, simulation-based workshops for healthcare professionals have been shown to enhance not only technical skills and self-efficacy but also teamwork and crisis management abilities in realistic emergency scenarios (49). In our study, this knowledge acquisition was reflected in practical performance, as 95.1% of beekeepers correctly used the training device within the required one-minute time frame. This emphasizes the program’s success not only in enhancing theoretical knowledge but also in developing the necessary practical skills for responding effectively to anaphylaxis.

Our findings are consistent with studies highlighting the importance of targeted health education in the context of anaphylaxis prevention and management. A study from Germany demonstrated that a structured 90-min educational intervention for patients with insect venom allergy significantly improved both their confidence in using emergency medication and their ability to manage an acute sting reaction (50). Comparable results were reported in research on an educational program for teachers and educators (23), which shows positive effects on their knowledge and preparedness to act in the case of allergic reactions. Brockow et al. (51) also note that educational programs play a key role in improving preparedness for managing anaphylaxis. Including high-risk groups for anaphylaxis in health education programs is essential to provide them with the skills necessary for early recognition and effective response to anaphylactic reactions. This confirms the need for broader implementation of educational strategies that address the specific needs of various professional groups and reduce the risk of serious health complications due to anaphylaxis. Although many studies recommend education as part of anaphylaxis prevention or management among beekeepers (5, 9, 15, 20), our work makes a distinct contribution by focusing on the development and evaluation of a dedicated health education program for this high-risk population.

In addition to traditional approaches, digital technologies are increasingly being explored as innovative tools for anaphylaxis education. For example, a study from Spain showed that an eight-week online training program for school staff significantly improved their knowledge, self-efficacy, and preparedness to manage food allergies and anaphylaxis, demonstrating the effectiveness of information and communication technology-based interventions as scalable and accessible educational solutions (22). Similarly, the AllergyAware e-learning course in Canada achieved high participant satisfaction, excellent post-course quiz results, and increased confidence in managing anaphylaxis, further highlighting the potential of digital education as an effective and scalable tool (52). Moreover, a mobile web-based educational program for parents of school-aged children with food allergies demonstrated improvements in knowledge, self-efficacy, and practical skills for managing food allergies and anaphylaxis, reinforcing the value of online approaches for targeted educational interventions (53). In our study, we retained a traditional, in-person approach, as the program is newly developed and aimed at beekeepers of different age groups. This ensured that the education remained accessible to all participants, regardless of their familiarity with digital technologies.

To evaluate the effectiveness of the educational program, the timing of the knowledge assessment was carefully considered. The decision regarding the knowledge assessment interval was based on research indicating that knowledge transfer within the first month after training is a reliable predictor of long-term knowledge retention (54). Furthermore, Brown et al. (55) state that a 3 to 6 week interval between tests improves the validity of results by reducing the likelihood that participants merely recall their previous answers. To further ensure the reliability of the results, we conducted the knowledge test with a 5 week interval between the pre-test and post-test, thereby preventing the learning effect from influencing the results.

However, researches also indicates that knowledge and skills acquired through short-term educational interventions may decline over time (56), meaning that the long-term impact on actual responses in crisis situations could not be fully assessed. For example, approximately one-third of AAI users no longer know how to correctly use the device after 1 year, and this proportion drops to only 18.6% after 2 years (57). Therefore, an important direction for future research is to evaluate the long-term impact of the educational program, including knowledge retention, potential behavioral changes, and the effect on the incidence of severe anaphylactic reactions. Such follow-up studies could provide valuable insights into the sustainability and real-world effectiveness of the intervention.

Building on the findings from our study, which highlighted how knowledge acquisition is connected to practical skills, it is important to incorporate behavioral theories and models into the development of educational programs (58). Theories explore how personal factors, like beliefs about risk and motivation, along with broader social, cultural, and economic influences, shape an individual’s health decisions. Using behavioral models in health interventions helps analyze these factors and develop targeted strategies for changing behavior, based on scientific insights, to improve health outcomes (59, 60). They are key to the development of effective health educational programs (61). In the development of the health education program, we considered key concepts from two important models: the Health Belief Model by Irwin M. Rosenstock, which emphasizes the impact of perceiving the severity of a disease and the benefits of actions on adopting preventive behaviors, and the Health Promotion Model by Nola J. Pender, which focuses on a positive approach to health and includes personal goals, health perception, and the interaction between personal and environmental factors (62–64). This allowed us to create a comprehensive approach to education that encourages understanding and implementation of health-promoting behaviors among beekeepers. In our study, we investigated beekeepers’ belief in their ability to act correctly in the event of anaphylaxis, their motivation for education, their perception of its importance, and their opinions on the impact of gender or age on beekeepers’ learning and response actions. The results showed that beekeepers believe they have gained knowledge on recognizing and responding to anaphylaxis through education. They also consider such education to be crucial. The belief that the educational content is important can enhance individuals’ motivation, which is a key factor in the success of health education programs (65, 66). In our study, we therefore explored how beekeepers’ perceptions of the importance of such education may influence their motivation to engage and apply the knowledge in practice. Furthermore, they view these educational programs as important for safety. However, they expressed the opinion that gender or age within their community does not affect their perception of risk, preparedness to act in the event of anaphylaxis, or the acquisition of knowledge. The factors of gender and age were important for us, as other studies have shown that men are at a higher risk for more severe anaphylaxis compared to women (67, 68). This helps us to gain a better understanding of how demographic differences influence the perception of risk and readiness to act. We acknowledge that gender-related differences observed in our study should be interpreted with caution. Our research does not rely on gender stereotypes, but aims to contribute to understanding factors that can influence preventive approaches. These findings may reflect social or cultural constructs rather than inherent biological differences. In line with the recommendations of the Task Force on Equality and the Gender-Net Plus Workshop (69), we emphasize the importance of critically re-evaluating such results in future studies to avoid gender-stereotype-based interpretations.

These findings allow for a comprehensive interpretation of the program’s outcomes. The results in participants’ knowledge, attitudes, and practical skills clearly reflect the intended effects of the educational program. Interpreted through the cognitive, affective, and conative dimensions of Bloom’s taxonomy (70, 71) most participants achieved adequate knowledge (≥80% correct answers) and expressed positive attitudes toward the importance of education, with age and gender, in their view, having no significant impact on their motivation to acquire knowledge or respond appropriately in case of anaphylaxis. In this study, Bloom’s taxonomy served as a conceptual framework to organize and interpret results related to knowledge acquisition, attitudes, and practical skills, without assessing all six levels of the cognitive domain or broader pedagogical applications. This framework offers an integrated perspective on participants’ understanding, motivation, and practical readiness to respond to anaphylaxis, and serves as a basis for future educational interventions and research in occupational allergy prevention.

## 5 Limitations

This study has certain limitations that should be taken into account when interpreting the findings. The BAALQ proved to be a suitable tool for assessing recognition of anaphylaxis symptoms and appropriate actions, but it does not include questions on awareness of risk factors. The relatively small pilot sample for the RAAS (*n* = 59) is below the ideal size suggested by Bonett’s formula but sufficient for a half-width of ±0.042 and consistent with pilot study recommendations, providing a reliable preliminary estimate of internal consistency. The knowledge assessment was conducted 5 week after the educational intervention, which does not allow for an evaluation of long-term knowledge retention or its impact on real-life behavior. Understanding whether beekeepers maintain their knowledge over time and whether their ability to respond appropriately to anaphylaxis improves with continued practice and experience remains an important area for further investigation. Future studies should include longitudinal assessments at various time points, such as 1 year after training, to provide deeper insight into knowledge retention and confidence levels. Another limitation is that the study sample included only Slovenian beekeepers, which means the results should be interpreted within the specific social, cultural, and healthcare context of the country. Factors such as access to medical care, national education programs, and cultural perceptions of risk may influence how applicable these findings are to other populations. Expanding research to include different geographical and cultural settings would offer a broader perspective on the educational program’s effectiveness. Exploring its adaptation for other high-risk groups, such as farmers, forestry workers, or individuals frequently exposed to insect stings, could further enhance its impact. In addition, beekeepers with a history of anaphylaxis were not included in this study; future studies will aim to include all beekeepers and their relatives, including those with a history of allergy, to further validate the educational program.

While these limitations should be acknowledged, they do not detract from the study’s significance. Instead, they emphasize the need for further research to refine educational strategies and improve anaphylaxis prevention efforts among high-risk groups.

## 6 Conclusion

A key finding of our study is that even individuals who have never experienced anaphylaxis can be trained to recognize its signs and respond correctly. This highlights the importance of broader implementation of educational initiatives tailored to the specific needs of diverse professional groups, ultimately reducing the risk of severe health complications related to anaphylaxis. Based on these outcomes, it is important that educational programs are tailored to specific groups and adapted to their unique characteristics, and it is recommended that they be regularly updated to ensure the sustainability and reinforcement of knowledge and skills. A longer follow-up period, such as 1 year after training, would allow for a more precise analysis of knowledge and skill retention, which are crucial for effective action in the event of an anaphylactic reaction. Such longitudinal studies could contribute to a more comprehensive understanding of the long-term effects of educational programs on individuals’ preparedness in crisis situations.

## Data Availability

The original contributions presented in the study are included in the article/supplementary material, further inquiries can be directed to the corresponding author.

## References

[1] Dribin TE Muraro A Camargo CA Jr Turner PJ Wang J Roberts G . Anaphylaxis definition, overview, and clinical support tool: 2024 consensus report—a GA2LEN project. J Allergy Clin Immunol. (2025) 156:406–417.e6. doi: 10.1016/j.jaci.2025.01.021, PMID: 39880313 PMC12301991

[2] Muraro A Worm M Alviani C Cardona V DunnGalvin A Garvey LH . EAACI guidelines: anaphylaxis (2021 update). Allergy. (2022) 77:357–77. doi: 10.1111/all.15032, PMID: 34343358

[3] Chatain C Sedillot N Thomas M Pernollet M Bocquet A Boccon-Gibod I . Fatal hymenoptera venom anaphylaxis by undetected clonal mast cell disorder: a better identification of high risk patients is needed. Rev Med Interne. (2021) 42:869–74. doi: 10.1016/j.revmed.2021.08.005, PMID: 34776279

[4] Feás X Vidal C Remesar S. What we know about sting-related deaths? Human fatalities caused by hornet, wasp and bee stings in Europe (1994–2016). Biology. (2022) 11:282. doi: 10.3390/biology11020282, PMID: 35205148 PMC8869362

[5] Carli T Locatelli I Košnik M Kukec A. The prevalence of self-reported systemic allergic reaction to Hymenoptera venom in beekeepers worldwide: a systematic literature review and Meta-analysis. Zdr Varst. (2024) 63:152–9. doi: 10.2478/sjph-2024-0020, PMID: 38881633 PMC11178027

[6] Burzyńska M Piasecka-Kwiatkowska D. A review of honeybee venom allergens and allergenicity. Int J Mol Sci. (2021) 22:8371. doi: 10.3390/ijms22168371, PMID: 34445077 PMC8395074

[7] Arcangeli G Traversini V Tomasini E Baldassarre A Lecca LI Galea RP . Allergic anaphylactic risk in farming activities: a systematic review. Int J Environ Res Public Health. (2020) 17:4921. doi: 10.3390/ijerph17144921, PMID: 32650469 PMC7399996

[8] Demirkale ZH Yücel E Çimen SS Süleyman A Özdemir C Kara A . Venom allergy and knowledge about anaphylaxis among beekeepers and their families. Allergol Immunopathol. (2020) 48:640–5. doi: 10.1016/j.aller.2020.01.008, PMID: 32460992

[9] Ediger D Terzioglu K Ozturk RT. Venom allergy, risk factors for systemic reactions and the knowledge levels among Turkish beekeepers. Asia Pac Allergy. (2018) 8:e15. doi: 10.5415/apallergy.2018.8.e15, PMID: 29732291 PMC5931922

[10] Müller UR. Bee venom allergy in beekeepers and their family members. Curr Opin Allergy Clin Immunol. (2005) 5:343–7. doi: 10.1097/01.all.0000173783.42906.95, PMID: 15985817

[11] Stanhope J Carver S Weinstein P. Health outcomes of beekeeping: a systematic review. J Apic Res. (2017) 56:100–11. doi: 10.1080/00218839.2017.1291208

[12] Moffitt JE Golden DB Reisman RE Lee R Nicklas R Freeman T . Stinging insect hypersensitivity: a practice parameter update. J Allergy Clin Immunol. (2004) 114:869–86. doi: 10.1016/j.jaci.2004.07.046, PMID: 15480329

[13] Bilò BM Bonifazi F. Epidemiology of insect-venom anaphylaxis. Curr Opin Allergy Clin Immunol. (2008) 8:330–7. doi: 10.1097/ACI.0b013e32830638c5, PMID: 18596590

[14] Worm M Höfer V Dölle-Bierke S Bilo MB Hartmann K Sabouraud-Leclerc D . Occupational anaphylaxis—data from the anaphylaxis registry. Allergy. (2024) 79:702–10. doi: 10.1111/all.15974, PMID: 38093663

[15] Richter AG Nightingale P Huissoon AP Krishna MT. Risk factors for systemic reactions to bee venom in British beekeepers. Ann Allergy Asthma Immunol. (2011) 106:159–63. doi: 10.1016/j.anai.2010.11.005, PMID: 21277518

[16] Bilò MB Antonicelli L Bonifazi F. Honeybee venom immunotherapy: certainties and pitfalls. Immunotherapy. (2012) 4:1153–66. doi: 10.2217/imt.12.113, PMID: 23194365

[17] Münstedt K Wrobel D Kalder M. Efficacy of venom immunotherapy in beekeepers. J Investig Allergol Clin Immunol. (2010) 20:58–62. 20232774

[18] Rijavec M Inkret J Bidovec-Stojković U Carli T Frelih N Kukec A . Fatal Hymenoptera venom–triggered anaphylaxis in patients with unrecognized clonal mast cell disorder—is mastocytosis to blame? Int J Mol Sci. (2023) 24:16368. doi: 10.3390/ijms242216368, PMID: 38003556 PMC10671356

[19] Treudler R Worm M Bauer A Dickel H Heine G Jappe U . Occupational anaphylaxis: a position paper of the German Society of Allergology and Clinical Immunology (DGAKI). Allergol Select. (2024) 8:407–24. doi: 10.5414/ALX02543E, PMID: 39659712 PMC11629776

[20] Sato K Hirata H Tatewaki M Shiromori S Souma R Satoh H . Emergency treatment of anaphylaxis in Japanese beekeepers. J Agromedicine. (2020) 25:153–7. doi: 10.1080/1059924X.2019.1674229, PMID: 31566096

[21] Polloni L Baldi I Amadi M Tonazzo V Bonaguro R Lazzarotto F . Management of children with food-induced anaphylaxis: a cross-sectional survey of parental knowledge, attitude, and practices. Front Pediatr. (2022) 10:886551. doi: 10.3389/fped.2022.886551, PMID: 35664871 PMC9160827

[22] Poza-Guedes P González-Pérez R. Implementing information and communication technology education on food allergy and anaphylaxis in the school setting. Clin Transl Allergy. (2021) 11:e12039. doi: 10.1002/clt2.12039, PMID: 34262693 PMC8254581

[23] Devetak I Devetak SP Vesel T. Future teachers' attitudes and knowledge regarding the Management of the Potential Students' life-threatening allergic reactions in Slovenian schools. Zdr Varst. (2018) 57:124–32. doi: 10.2478/sjph-2018-0016, PMID: 29983778 PMC6032179

[24] Shang Z. Use of Delphi in health sciences research: a narrative review. Medicine. (2023) 102:e32829. doi: 10.1097/MD.0000000000032829, PMID: 36800594 PMC9936053

[25] Makhmutov R. The Delphi method at a glance. Pflege. (2021) 34:221. doi: 10.1024/1012-5302/a000812, PMID: 34292075

[26] Močnik T Ličen S Zidarn M Prosen M. Exploring beekeepers’ experiences and perceptions of anaphylaxis risks: a qualitative study to inform targeted health education programs. Healthcare. (2024) 12:2569. doi: 10.3390/healthcare12242569, PMID: 39765996 PMC11727721

[27] Hasson F Keeney S McKenna H. Research guidelines for the Delphi survey technique. J Adv Nurs. (2000) 32:1008–15. doi: 10.1046/j.1365-2648.2000.t01-1-01567.x, PMID: 11095242

[28] Vaismoradi M Turunen H Bondas T. Content analysis and thematic analysis: implications for conducting a qualitative descriptive study. Nurs Health Sci. (2013) 15:398–405. doi: 10.1111/nhs.12048, PMID: 23480423

[29] Yusoff MSB. ABC of content validation and content validity index calculation. Educ Med J. (2019) 11:49–54. doi: 10.21315/eimj2019.11.2.6

[30] Noushad B Van Gerven PW De Bruin AB. Twelve tips for applying the think-aloud method to capture cognitive processes. Med Teach. (2024) 46:892–7. doi: 10.1080/0142159X.2023.2289847, PMID: 38071621

[31] Meadows K. Cognitive interviewing methodologies. Clin Nurs Res. (2021) 30:375–9. doi: 10.1177/10547738211014099, PMID: 33998325

[32] Taber KS. The use of Cronbach’s alpha when developing and reporting research instruments in science education. Res Sci Educ. (2018) 48:1273–96. doi: 10.1007/s11165-016-9602-2

[33] Ekolu SO Quainoo H. Reliability of assessments in engineering education using Cronbach’s alpha, KR and split-half methods. Glob J Eng Educ. (2019) 21:24–9.

[34] Bujang MA Omar ED Foo DHP Hon YK. Sample size determination for conducting a pilot study to assess reliability of a questionnaire. Restor Dent Endod. (2024) 49:e3. doi: 10.5395/rde.2024.49.e3, PMID: 38449496 PMC10912549

[35] Kishore K Jaswal V Kulkarni V De D. Practical guidelines to develop and evaluate a questionnaire. Indian Dermatol Online J. (2021) 12:266–75. doi: 10.4103/idoj.IDOJ_674_20, PMID: 33959523 PMC8088187

[36] Leung ASY Cheng NS Cheng JWCH Pun J Leung TF. Development and validation of assessment tools for food allergy–related knowledge and management confidence. J Allergy Clin Immunol Glob. (2023) 2:100098. doi: 10.1016/j.jacig.2023.100098, PMID: 37779529 PMC10509832

[37] Mundfrom DJ Shaw DG Ke TL. Minimum sample size recommendations for conducting factor analyses. Int J Test. (2005) 5:159–68. doi: 10.1207/s15327574ijt0502_4

[38] Polit DF Beck CT. Essentials of nursing research: Appraising evidence for nursing practice. 9th ed. Philadelphia: Wolters Kluwer Health (2018).

[39] Sharma S. Nursing research and statistics. 5th ed. [e-book]. New Delhi: Elsevier Health Sciences (2025).

[40] Shrestha N. Factor analysis as a tool for survey analysis. Am J Appl Math Stat. (2021) 9:4–11. doi: 10.12691/ajams-9-1-2

[41] Ravid R. Practical statistics for educators. 7th ed. Lanham: Rowman & Littlefield (2024).

[42] Heo M Kim N Faith MS. Statistical power as a function of Cronbach alpha of instrument questionnaire items. BMC Med Res Methodol. (2015) 15:86. doi: 10.1186/s12874-015-0070-6, PMID: 26467219 PMC4606843

[43] Bonett DG. Sample size requirements for testing and estimating coefficient alpha. J Educ Behav Stat. (2002) 27:335–40. doi: 10.3102/10769986027004335

[44] Hair JF Jr Black WC Babin BJ Anderson RE. Multivariate Data Analysis. 7th ed. Harlow, Essex: Pearson Education (2014).

[45] Bandalos DL Finney SJ. Factor analysis: exploratory and confirmatory In: Hancock GR Stapleton LM Mueller RO, editors. The reviewer’s guide to quantitative methods in the social sciences. London: Routledge (2018). 98–122.

[46] Chow Wei L Yazid MB Norhayati MN Md Noh AY Rahman A. Patient ability to use old versus new/modified model adrenaline autoinjection emergency medical devices for anaphylaxis in prehospital setting: a systematic review and meta-analysis. Healthcare. (2022) 10:183. doi: 10.3390/healthcare10020183, PMID: 35206798 PMC8872424

[47] Kim YH Kim AL. Evaluating standardized job competencies for managing students at risk for anaphylaxis in elementary school nurses. Healthcare. (2023) 11:2102. doi: 10.3390/healthcare11142102, PMID: 37510543 PMC10379963

[48] Sasaki K Sugiura S Matsui T Nakagawa T Nakata J Kando N . A workshop with practical training for anaphylaxis management improves the self-efficacy of school personnel. Allergol Int. (2015) 64:156–60. doi: 10.1016/j.alit.2014.10.005, PMID: 25838091

[49] Chong M Pasqua D Kutzin J Davis-Lorton M Fonacier L Aquino M. Educational and process improvements after a simulation-based anaphylaxis simulation workshop. Ann Allergy Asthma Immunol. (2016) 117:432–3. doi: 10.1016/j.anai.2016.07.025, PMID: 27522110

[50] Schoeben LS Mohr N Bubak C Schmieder A Schaarschmidt ML. Effects of a 90-min educational intervention for patients with insect venom allergy: a prospective controlled pilot study. Allergy Asthma Clin Immunol. (2021) 17:22. doi: 10.1186/s13223-021-00524-7, PMID: 33632327 PMC7905619

[51] Brockow K Schallmayer S Beyer K Biedermann T Fischer J Gebert N . Effects of a structured educational intervention on knowledge and emergency management in patients at risk for anaphylaxis. Allergy. (2015) 70:227–35. doi: 10.1111/all.12548, PMID: 25407693

[52] Sharma B Ayers S Huang J Gerdts J Waserman S Levinson AJ. Online food allergy and anaphylaxis education for school personnel is effective and scalable: experience with the allergyaware e-learning portal from 2015 to 2022. Allergy Asthma Clin Immunol. (2025) 21:30. doi: 10.1186/s13223-025-00977-0, PMID: 40619393 PMC12232682

[53] Kwen H Oh PJ. Development and evaluation of a mobile web-based food allergy and anaphylaxis management educational program for parents of school-aged children with food allergy: a randomized controlled trial. Asian Nurs Res. (2022) 16:265–74. doi: 10.1016/j.anr.2022.10.002, PMID: 36334689

[54] Axtell CM Maitlis S Yearta SK. Predicting immediate and longer-term transfer of training. Pers Rev. (1997) 26:201–13. doi: 10.1108/00483489710161413

[55] Brown G Irving E Keegan P. An introduction to educational assessment, measurement, and evaluation: Improving the quality of teacher-based assessment. 2nd ed. Auckland: Pearson Education (2008).

[56] Topal E Bakirtas A Yilmaz O Karagol IHE Arga M Demirsoy MS . When should we perform a repeat training on adrenaline auto-injector use for physician trainees? Allergol Immunopathol. (2014) 42:472–5. doi: 10.1016/j.aller.2013.07.008, PMID: 24176470

[57] Sirin Kose S Asilsoy S Tezcan D Al S Atay O Kangalli O . Is there an optimal training interval to improve the correct use of adrenaline auto-injectors? Int Arch Allergy Immunol. (2020) 181:136–40. doi: 10.1159/000504365, PMID: 31794965

[58] Marmot M Wilkinson RG. Social determinants of health. Oxford: Oxford University Press (2005).

[59] Rejeski WJ Fanning J. Models and theories of health behavior and clinical interventions in aging: a contemporary, integrative approach. Clin Interv Aging. (2019) 14:1007–19. doi: 10.2147/CIA.S206974 31213787 PMC6549388

[60] Raphael D. Social determinants of health: key issues and themes In: Raphael D Bryant T, editors. Social determinants of health: Canadian perspectives. 3rd ed. Toronto: Canadian Scholars’ Press (2016). 3–31.

[61] McKenzie JF Neiger BL Thackeray R. Planning, implementing and evaluating health promotion programs with navigate advantage access. 8th ed. Burlington, MA: Jones & Bartlett Learning (2022).

[62] Wilson JC Nola J. Pender: health promotion model In: Alligood MR, editor. Nursing theorists and their work. 10th ed [e-book]. St. Louis: Elsevier (2021). 320.

[63] Anuar H Shah SA Gafor H Mahmood MI Ghazi HF. Usage of health belief model (HBM) in health behavior: a systematic review. Malaysian J Med Health Sci. (2020) 16:2636–9346.

[64] Abraham C Sheeran P. The health belief model. In: Conner M. Norman P. (eds) Predicting health behaviour: Research and practice with social cognition models. 2. Maidenhead: Open University Press; 2005. 28–80.

[65] Michaelsen MM Esch T. Understanding health behavior change by motivation and reward mechanisms: a review of the literature. Front Behav Neurosci. (2023) 17:1151918. doi: 10.3389/fnbeh.2023.1151918, PMID: 37405131 PMC10317209

[66] Filgona J Sakiyo J Gwany DM Okoronka AU. Motivation in learning. Asian J Educ Soc Stud. (2020) 10:16–37. doi: 10.9734/AJESS/2020/v10i430273

[67] Höfer V Dölle-Bierke S Francuzik W Ruëff F Sabouraud-Leclerc D Treudler R . Fatal and near-fatal anaphylaxis: data from the European anaphylaxis registry and National Health Statistics. J Allergy Clin Immunol Pract. (2024) 12:e8:96–105. doi: 10.1016/j.jaip.2023.09.044, PMID: 37816460

[68] Carli T Locatelli I Košnik M Bevk D Kukec A. Epidemiology and risk factor analysis of systemic allergic reaction to bee venom in the Slovenian population of beekeepers. Zdr Varst. (2025) 64:40–8. doi: 10.2478/sjph-2025-0006, PMID: 39758088 PMC11694630

[69] Cederroth CR Earp BD Gómez Prada HC Jarach CM Lir SA Norris CM . Integrating gender analysis into research: reflections from the gender-net plus workshop. EClinicalMedicine. (2024) 74:102728. doi: 10.1016/j.eclinm.2024.102728, PMID: 39105192 PMC11299571

[70] Grebin N Grabovska S Karkovska R Vovk A. Applying Benjamin Bloom's taxonomy ideas in adult learning. J Educ Cult Soc. (2020) 11:61–72. doi: 10.15503/jecs2020.1.61.72

[71] Bloom BS Engelhart MD Furst EJ Hill WH Krathwohl DR. Handbook I: Cognitive domain. New York: David McKay (1956).

